# Investigating Microstructural Evolution and Its Influence on Tribological Behaviors of In-Situ Formed VCp Reinforced Iron-Based Composites with Variable Mn Content

**DOI:** 10.3390/ma15228158

**Published:** 2022-11-17

**Authors:** Pinghu Chen, Wenguang Zhao, Zhen Liu, Yun Zhang, Caifeng Weng, Ruiqing Li, Yong Chen

**Affiliations:** 1 College of Mechatronics & Control Engineering, Shenzhen University, Shenzhen 518060, China; 2 School of Advanced Materials, Peking University Shenzhen Graduate School, Shenzhen 518055, China; 3 School of Mechanical Engineering, Hunan University of Science and Technology, Xiangtan 411201, China; 4 State Key Laboratory of High Performance Complex Manufacturing, Light Alloys Research Institute, Central South University, Changsha 410083, China; 5 College of Mechanical Engineering, University of South China, Hengyang 421001, China

**Keywords:** vanadium carbide, iron-based composites, microstructural evolution, hardness, tribological behaviors

## Abstract

In this work, we fabricated VCp-reinforced iron-based composites (VCFCs) by adjusting the amount of Mn elements and investigated how the concentration of Mn affected the microstructural characteristics of Vanadium carbide (VC) and the texture of the iron matrix, and the influence of microstructure on tribological behaviors should be investigated. We demonstrated that VC changed from thick dendrite crystals (~50 μm) to tiny equiaxed crystals (~5 μm). Furthermore, the nucleation mechanism of VC also transformed from homogeneous nucleation to heterogeneous nucleation due to the lower Gibbs free energy of TiC and the tailoring effect of the Mn elements. In addition, γ-Fe in the FCC structure gradually increased and ascribed an increase of Mn content to the lower transformation temperature of martensite. Furtherly, particulate features and phase constitution could contribute to hardness and wear resistance. Higher hardness and excellent wear resistance occurred in the 3.0 Mn sample, which had a hardness of 869 HV and a wear rate of 1.77 × 10^−6^ mm^3^/(N·m). In addition, the adhesive wear could be the main wear mechanism in the 3.0 Mn sample, while the abrasive wear could be in the 4.5 Mn sample.

## 1. Introduction

Low energy consumption and environmental protection are advocated in today’s society. There are higher requirements for the service life of machinery components in mining, transportation, and civil engineering. Especially, some components employed in high load and friction environments, which were required to have excellent wear resistance. Hard particle (SiC, WC, and VC) strengthening technology holds technological promise for improving Fe-based alloys to suit practical applications in industrial, aerospace, and marine industries [1,2,3]. In general, the mechanical properties and wear resistance of hard particle-reinforced iron-based composites will deteriorate due to chemically non-uniform distribution, particle agglomeration, and poor wettability, preventing them from widespread application [4,5]. Fortunately, the mechanical properties and wear resistance of hard particle-reinforced iron-based composites could be improved through in situ reinforced hard particle (VC, TiC, and TiB_2_) doping due to the intrinsic properties of their endogenous particles and formation conditions [6,7]. Microstructural characteristics (shape, size, and distribution) and the wettability between the particles and Fe-based materials could be adjusted by the choice of chemical composition, preparation technology, and pre-/post-treatment [8,9,10,11,12].

VC has a stable structure and a high hardness value of approximately 2460–3150 HV [13]. More importantly, it could form spontaneously on account of a lower Gibbs free energy (∆G) under the process of solidification [14]. In the past 20 years, some studies reported the influence of chemical composition and subsequent heat treatment on the microstructure, mechanical properties, and wear resistance [15]. VC will form martensite (Ms) and austenite matrices in situ, improving the wear resistance due to the uniform dispersion of VC after subsequent heat treatment of quenching and tempering [16]. Li, X. indicated that a novel in situ VC-reinforced Fe-based composites could be designed by adding vanadium elements to 35CrMo LMD materials, and excellent wear resistance was obtained on account of uniform dispersion and fine-grain strengthening caused by in situ VC [17]. The VC particles formed in situ in Fe-13Mn and Fe-13Mn-3W alloys, and the strength, tensile properties, and work hardening rate were reinforced due to the addition of W elements. Meanwhile, VC has been shown to contribute to excellent wear resistance [18]. Nevertheless, the excessive addition of tungsten will weaken the wear resistance of Fe-based, wear-resistant composites because the distribution homogeneity and volume fraction of in situ VC will be reduced with the formation of the W_3_C phase [19]. A novel particle-reinforced, Fe-based, wear-resistant composite with in situ VC was designed. Multistage heat treatments were employed to adjust the microstructure, mechanical properties, and wear resistance [20,21,22]. Excellent wear resistance could be attributed to the uniform dispersion of in situ VC with a high proportion, and good mechanical properties were caused by a certain amount of retained austenite. This retained austenite could be transformed into martensite under abrasion, thus enhancing further wear resistance [23,24]. In addition, the nucleation and growth of VC were studied experimentally, and the quantity and morphology of the carbides could be controlled by a small amount of Ti elements [25]. However, the influence of Mn elements on the nucleation and growth of VC has rarely been reported.

In this study, we applied the sand cast technique to fabricate VCp-reinforced iron-based composites by tailoring the amount of Mn elements to elucidate the specific role of the action mechanism of Mn elements on the nucleation and growth of VCs. Meanwhile, the influence of Mn content on hardness and tribological behaviors was explored systematically.

## 2. Materials and Methods

**Figure 1:**
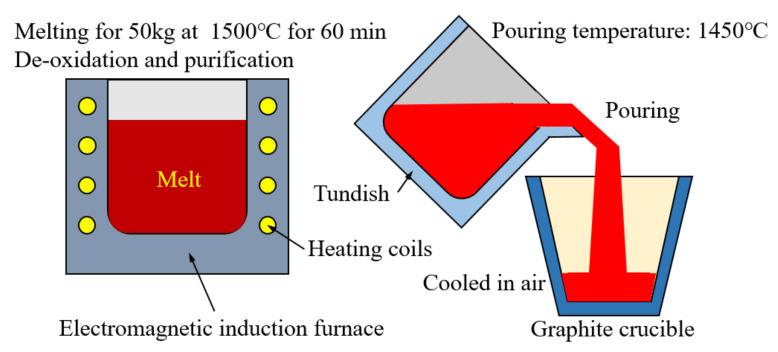
Schematic diagram of the casting process.

Three VCFCs with different Mn content were designed. Their chemical composition ratios are presented in Table 1. A 50 kg medium-frequency electromagnetic induction furnace was employed to melt three VCFCs. A schematic of the smelting-pouring-forming process is shown in Figure 1. During smelting, the melting temperature was set to 1500 °C. Ferrovanadium was added to improve the absorptivity of the vanadium element, and 1.5 wt.% pure titanium was added to improve the probability of heterogeneous nucleation of VC. Afterward, the molten iron was re-refined through the addition of 0.3 wt.% pure Al. Then, the purified molten iron at 1450 °C was poured into U-type molds and cooled in the open air.

The 10 mm × 10 mm × 5 mm specimens were cut from the ingots of three VCFCs, and all specimens were ground with metallographic 400#, 600#, 1000#, 1500#, 2000#, and 3000# sandpaper. Subsequently, a SiC polishing solution with 1.0 and 0.5 μm was adopted to polish all specimens. In addition, argon ion beam polishing was employed to remove the retained stress of the polished surface. The nanoindentation tests were conducted using a Hysitron TI 950 equipment with 8000 μN. Load-displacement curves and the corresponding nano hardness were obtained at the local region with the VC particle, Cr_7_C_3_ particle, and matrix. Microhardness was measured by a Vickers hardness tester (Micro Vickers HV-1000Z, MEGA INSTRUMENTS, Shanghai, China), operated at a load of 1000 gf with a duration time of 15 s. Five points were reported and average microhardness was calculated to be the final value. Besides that, a friction-wear machine (HT-1000, Zhongke Kaihua, Lanzhou, China) was employed to obtain tribological behaviors of friction coefficient and wear rate. The operation parameters included a friction speed of 500 r/min, friction force of 10 N, rotation radius of 1.5 mm, friction time of 60 min, and a Si_3_N_4_ counterpart. In addition, the average volume wear rate was calculated in the light of the equation w = (S·L)/(F·l), w was the worn rate, mm^3^/(N·m); S was the worn area, mm^2^; L was the circumference of the wear track, mm; F was friction force, N; l was the total distance, m.; where, the average worn area was quantified using a 3D laser confocal microscope (VK-X200 series, Keyence Corporation of America, Itasca, IL, USA) through four values obtained at the quartered position of the wear track.

A field emission scanning electron microscope (FE-SEM, Carl Zeiss, SUPRA^®^55, Oberkochen, Germany) was employed to characterize the shape, size, and distribution of the VC particles. Meanwhile, energy-dispersive X-ray spectroscopy (EDS) was employed to determine the elemental distribution. In addition, the textures of the three VCFCs were determined by high-energy X-ray diffraction (HE-XRD, Bruker, D8 discover, Billerca, MA, USA) with a Cu target (λ = 0.15418 nm), where the corresponding data were collected at 2θ = 20°–120° using a 0.05 step size. To obtain more detailed information on the textures and corresponding orientation, EBSD measurements were performed using a FEI SCIOS focused ion beam scanning electron microscope (SEM) with a Hikari camera (EDAX company, Santa Clara, CA, USA)and TSLOIM data-collection software (TexSem Laboratiries, Inc., Provo, UT, USA). The EBSD measurements were acquired using Oxford symmetry (Oxford instrument, Oxford, UK)with the following SEM and scanning parameters: 15 keV accelerating voltage, 12 Na probe current, 18 mm working distance, and a 30-nm step size. Data cleanup involved grain confidence-index standardization. The interfaces between the Fe matrix and carbide were examined using transmission electron microscopy (TEM: JEM-3200FS, JEOL, Tokyo, Japan) and scanning transmission electron microscopy (STEM). To prepare the site-specific samples for TEM analysis, the TEM foils were prepared directly from the interfacial region of interest by a focused-ion beam (FIB; SCIOS, gallium ion source).

## 3. Results

### 3.1. Microstructure and Phase Constitution

**Figure 2:**
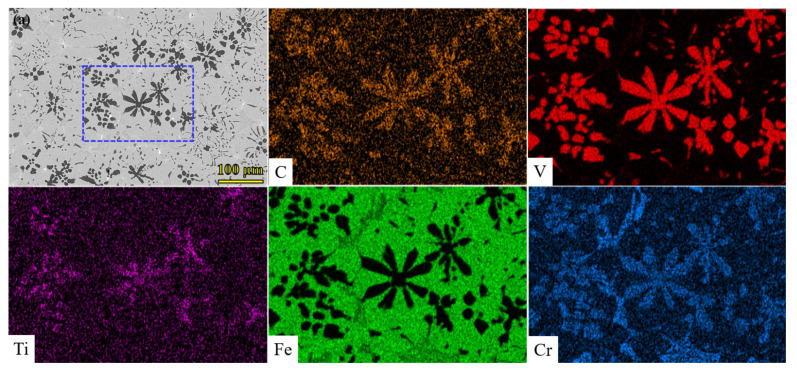

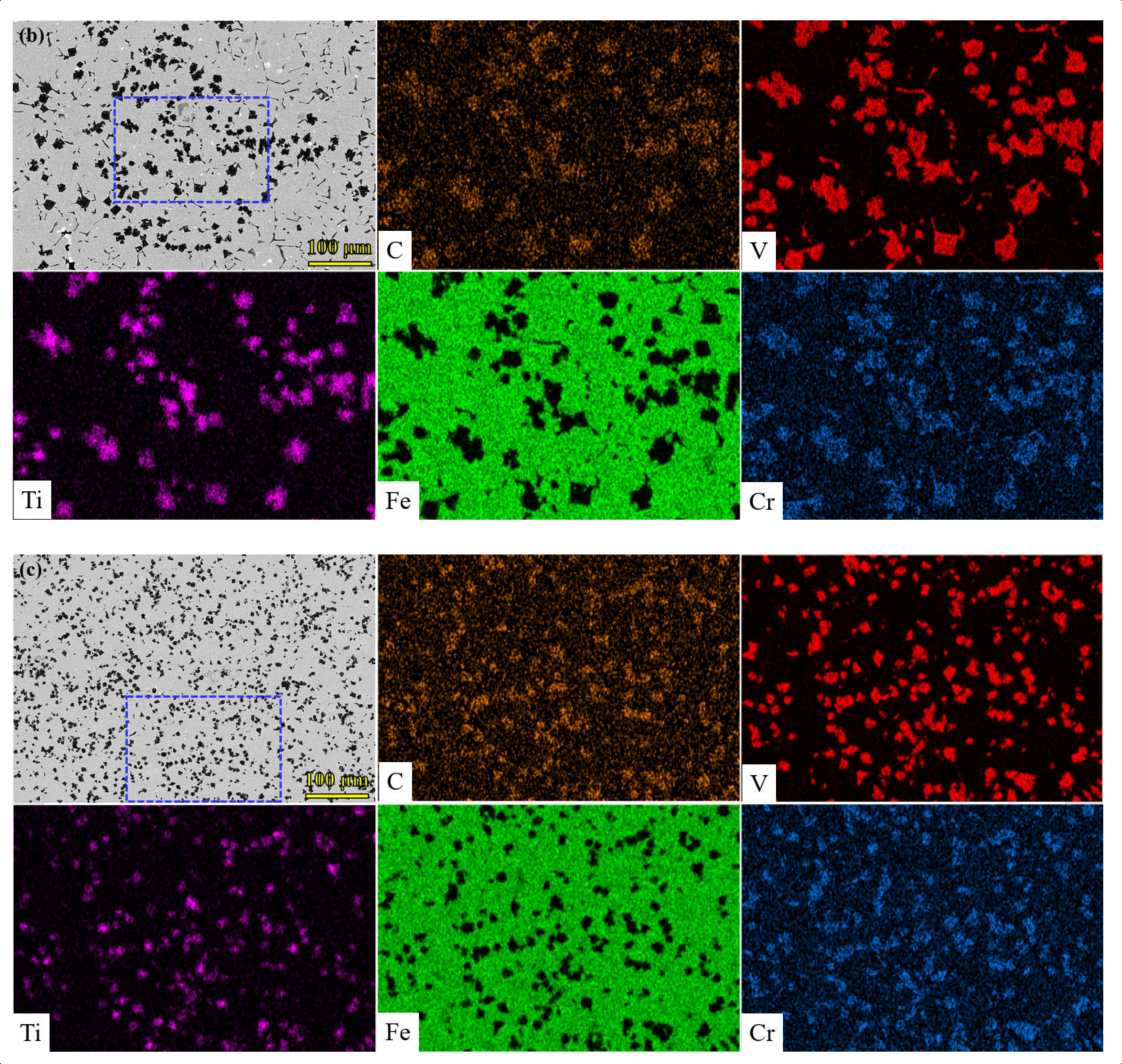
SEM images and EDS mapping of three samples. (**a**) 1.5 Mn; (**b**) 3.0 Mn; and (**c**) 4.5 Mn. The maps of elemental distribution is only presented within the blue frames.

The morphology features and composition distribution of the three samples are shown in Figure 2. A large amount of thick flower-petal particles with a size of approximately 50 μm is distributed inhomogeneously in the 1.5 Mn sample. Meanwhile, punctate (~5 μm), striped (thickness of 2–5 μm), and net-like carbide are distributed around the flower-petals particles, as shown in Figure 2a. For the 3.0 Mn sample, the thick flower-petals particles disappear, and relatively uniform block-like particles of approximately 10 μm are discovered in the iron matrix. Meanwhile, a small quantity of punctate and striped carbide is still found in the matrix (detailed information is presented in Figure 2b), but the net-like morphology is never discovered. Specifically, tiny carbides of block-like particles are distributed uniformly in the VCFCs without punctate and striped carbides (Figure 2c) when Mn content is increased to 4.5 wt.%. Therefore, with an increasing amount of Mn content, the shapes of the VC particles change from thick flower-petals particles to tiny block-like particles only 5 μm in size with 4.5 wt.% Mn content, and the size is 50 μm with 1.5 wt.% Mn content. In addition, the distribution of VC particles is more uniform when the Mn content is increased gradually. Combined with the chemical compositions of the three samples, it is obvious that the chemical compositions of the black particles mainly consist of V, C, Cr, and Ti elements, and additional elements of Mn, Si, and Mo are distributed uniformly in the matrix. Meanwhile, the Ti element appears in the same position as the V element, and its content is increased with increasing Mn content owing to a brighter color. The net-like precipitate is found in the 1.5 Mn sample, which mainly includes Cr and C elements.

**Figure 3:**
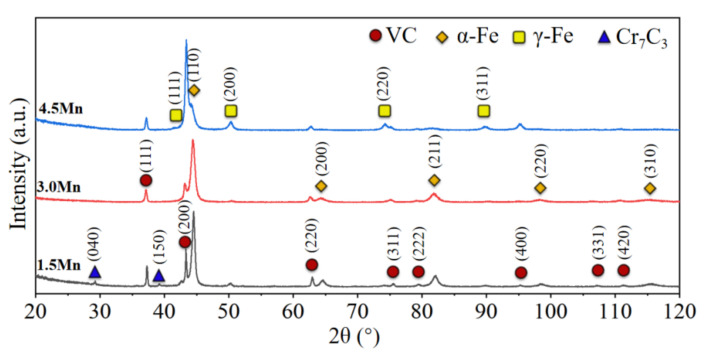
High-energy XRD patterns of three samples with different Mn content.

**Figure 4:**
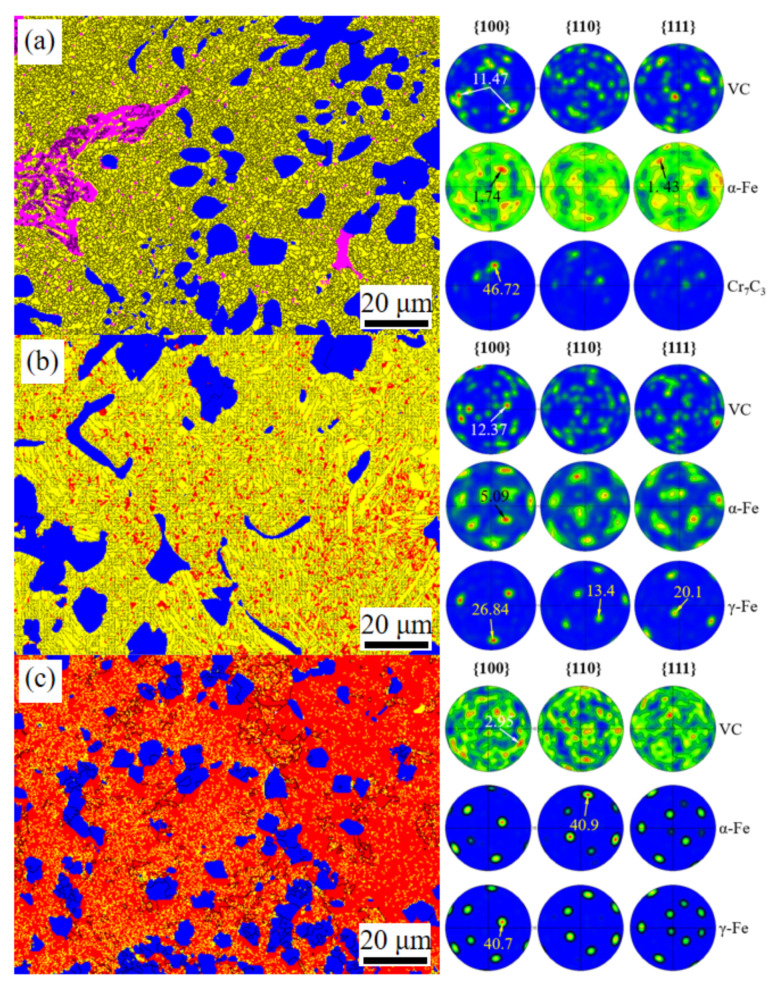
EBSD images and the corresponding pole figures of three samples with (**a**) 1.5 Mn; (**b**) 3.0 Mn; (**c**) 4.5 Mn.

In addition, a significant difference in the crystallization phases is caused by changes in Mn content in the VCFCs; XRD patterns are obtained as shown in Figure 3. The XRD patterns reveal that the VCFCs mainly consisted of VC and α-Fe with a BCC structure in the 1.5 Mn sample. Beyond that, a few Cr_7_C_3_ carbides are also discovered in the 1.5 Mn sample. Nevertheless, γ-Fe with an FCC structure is detected besides VC particles and α-Fe in the 3.0 Mn sample and 4.5 Mn sample. Combined with Figure 4, the main phase includes α-Fe with a BCC structure (yellow), flower-like VC (blue), and net-like Cr_7_C_3_ carbide [26] (pink) in the 1.5 Mn sample. Combined with the EBSD data, we found that the Cr_7_C_3_ phase has a maximum index in the {100} PF with an intensity of 46.72, indicating a remarkable orientation. Compared to the {100} PF, the intensities of the Cr_7_C_3_ texture index in the {110}, {111} PFs are relatively weaker, and the results imply that Cr_7_C_3_ grew mainly along the {100} orientation. The textures of VC and α-Fe are relatively random, and the corresponding maximum intensities of VC and α-Fe are 11.47 and 1.74, respectively, in the {100}, {110}, and {111} PFs. However, in the 3.0 Mn and 4.5 Mn samples, Cr_7_C_3_ carbide disappears when γ-Fe with an FCC structure (red) appears with increasing Mn content, as shown in Figure 4b,c. Similarly, the texture of VC appears to be nearly random, with a maximum intensity of 12.37 index in the {100} PF. Nevertheless, it is obvious that the intensity of the α-Fe texture in the {100}, {110}, {111} PF becomes increasingly stronger with increasing Mn content, and the value reaches 40.9 in the {110} PF. In addition, the texture of γ-Fe shows the same tendency, in terms of intensity, and grows mainly along the {100} orientation.

**Figure 5:**
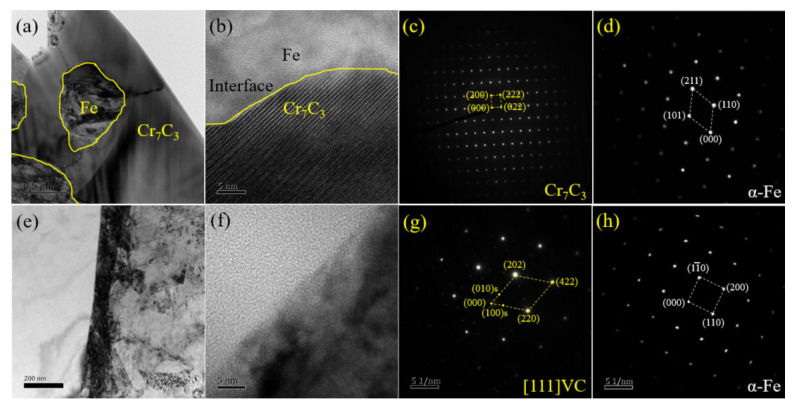
TEM image and electron-diffraction spot in the representative 1.5 Mn sample: (**a**) SEM image of Cr_7_C_3_ carbide; (**b**) HR-TEM image of the interface between α-Fe and Cr_7_C_3_ carbide; (**c**) ED of Cr_7_C_3_; and (**d**) ED of α-Fe. (**e**) SEM image and (**f**) HR-TEM image in the interface between α-Fe and VC; (**g**) ED of VC in the [111] zone; and (**h**) ED of α-Fe.

**Figure 6:**
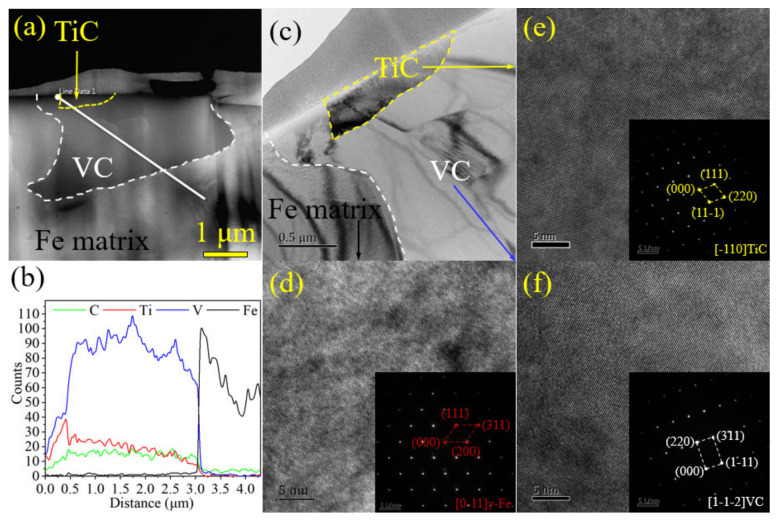
The HR-TEM image and electron-diffraction spot of the rich Ti region throughout the VC in the representative 4.5 Mn sample: (**a**) SEM image and composition distribution in the line-scan results; (**b**) magnified image in the (**a**); and (**c**–**f**) HR-TEM image and their corresponding electron-diffraction spots of TiC, Fe, and VC.

In order to further identify the crystalline structure and nucleation-growth mechanism, microstructure, high-resolution TEM images, and the electron diffraction spot of two typical samples are displayed in Figure 5 and Figure 6. Figure 5a indicates that Cr_7_C_3_ has a continuous net-like structure and Fe is surrounded by rich-Cr carbide in the 1.5 Mn sample. The HR-TEM image of the interface between the Fe matrix and the Cr_7_C_3_ carbide is shown in Figure 5b. Meanwhile, the zone axis of [01–1] Cr_7_C_3_ is accompanied by the (101), (110), and (211) crystal planes in the Fe phase with a BCC structure, as shown in Figure 5c,d. Figure 5e shows the TEM image in the interface between the Fe matrix and VC carbide, which has an obvious transition region with a width of 100 nm. Of note, VC is composed of VC (as shown by the strong electron-diffraction spot) and accompanied by V_6_C_5_ (depicted by the weak electron-diffraction spot marked with an “s”) [1], as shown in Figure 5g,h. Rich-Ti carbide is never observed in the XRD, EBSD, and TEM results for the 1.5 Mn sample. However, it is obvious that the rich Ti compound is in the center of VC in the 3.0 Mn and 4.5 Mn samples (Figure 2). Therefore, FIB is employed to cut a TEM sample from the 4.5 Mn sample. The TEM image and composition distribution from the line-scan results throughout the VC are depicted in Figure 6a,b. Note that a 1 μm nucleus with rich Ti elements is captured within the coarse grains of the VC phase. The Fe, TiC, and VC phases are identified with the zone axis of [0–11] γ-Fe, [−110] TiC, and [1–1–2] VC, as shown in Figure 6d–f.

### 3.2. Hardness

**Figure 7:**
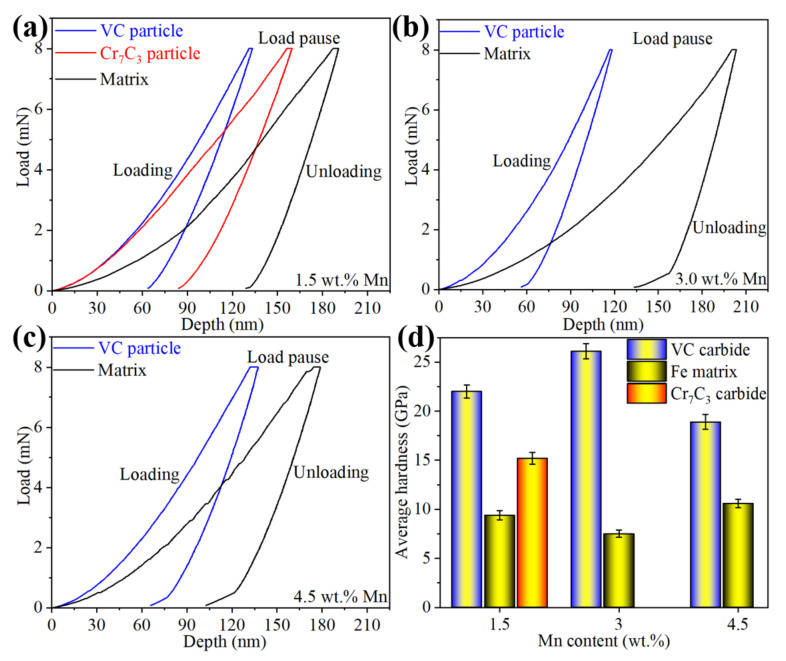
Load-displacement curves (**a**–**c**) and nano hardness responses (**d**) of three samples with different local regions.

**Figure 8:**
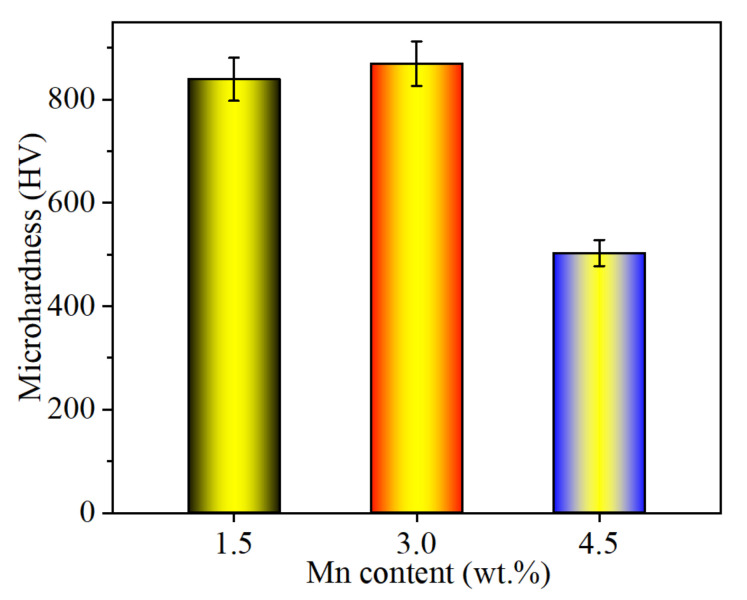
Microhardness—three samples.

The microstructure can contribute to physical properties. Nanoindentation load-displacement curves of carbides and Fe matrix for three samples are presented in Figure 7a–c. Note that the depth of VC carbide is shallower than that of Cr_7_C_3_ carbide, and the depth of the Fe matrix is the minimum in the same sample. However, for different samples, the depth of the VC carbide is increased at first and then decreases with increasing Mn content, and a contradictory trend exists in the matrix. It is indicated that the lightest depth has the highest hardness when the largest depth contributes to the lowest hardness, as shown in Figure 7d. Nano hardness of the VC carbide in the 3.0 Mn sample is the highest compared with the others owing to higher-ratio TiC carbide with a hardness of 31.4 GPa [27]. However, higher-content γ-Fe and smaller-scale VC particles can contribute to lower hardness because the whole subsidence of the VC particle is caused by loading with 8 mN. At the same time, the dense distribution of tiny VC carbides can cause higher hardness in the matrix. In addition to nano hardness in different phases, the average microhardness of three samples is obtained, as shown Figure 8. The results could be in accordance with nano hardness, their values of the 1.5 Mn, 3.0 Mn, and 4.5 Mn samples are 839, 869, and 503 HV, respectively. It is well known that hardness is an important factor in tribological behaviors. In general, higher hardness contributes to excellent wear resistance. However, friction pairs of engineering components should not be abraded seriously in the service process. Consequently, a relatively low friction coefficient is another important factor for wear resistance.

### 3.3. Tribological Behaviors

**Figure 9:**
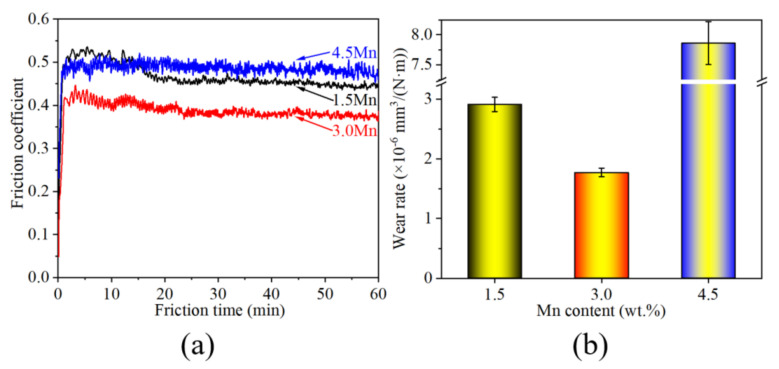
Friction coefficient (**a**) and wear rate (**b**) of three samples.

Figure 9 shows the friction coefficient and average volume wear rate of three samples. A higher friction coefficient of 1.5 Mn and 3.0 Mn samples occur at the seedling stage, but a small decrease exists at the steady state. This phenomenon never appears in the 4.5 Mn sample. The average friction coefficient at the steady state was calculated, and their values for 1.5 Mn, 3.0 Mn, and 4.5 Mn samples are 0.45, 0.38, and 0.48, respectively. The minimum value exists in the 3.0 Mn sample. The aforementioned results indicate that the wear resistance of the 3.0 Mn sample is better than other samples owing to higher hardness and lower friction coefficient. As in Figure 9b, the wear rate exhibits a decrease first and then increases. Their values are 2.91 × 10^−6^ mm^3^/(N·m), 1.77 × 10^−6^ mm^3^/(N·m), and 7.86 × 10^−6^ mm^3^/(N·m), respectively. This indicates that excellent wear resistance with the minimum wear rate occurs in the 3.0 Mn sample, and its relative wear resistance is 64.4% and 344.1% higher than that of the 1.5 Mn and 4.5 Mn samples, respectively.

## 4. Discussion

### 4.1. Influence Mechanism of Mn Content on Microstructure

Microstructural evolution can be attributed to the change in Mn content. Titanium has a high melting point of 1668 °C, with good chemical activity, and can be considered a heterogeneous nucleation site in particle-reinforced iron-based composite and Al alloys [28,29]. However, based on the data in Table 1, only the content of Mn and Fe elements changed, and the others did not change. We found that the content of Mn elements is the main factor in the difference in the carbide features (shape, size, and distribution) and Fe phase.

**Figure 10:**
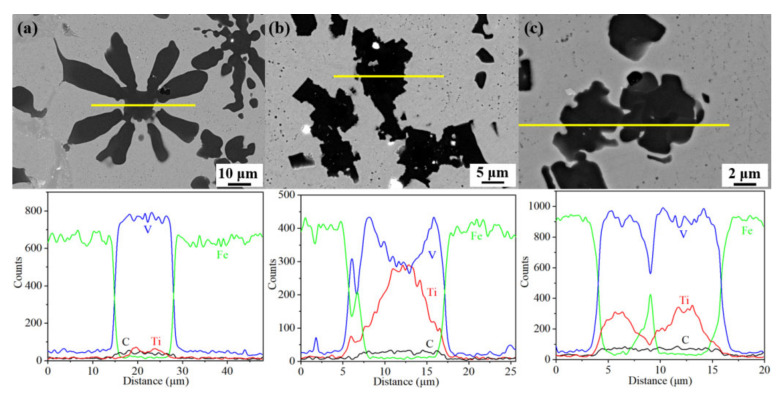
SEM image and the corresponding EDS composition in line scanning: (**a**) 1.5 Mn sample; (**b**) 3.0 Mn sample; and (**c**) 4.5 Mn sample.

Firstly, the nucleation mode is influenced by the turning of the Mn content. Mn had an incomplete outer electron layer to cause electronic redistribution and increases the bonding force between atoms when entering the lattice. This results in inhibiting the diffusion of other elements [30]. With a low content of Mn elements, the other alloying elements show a relatively rapid diffusion rate, where VC and Cr_7_C_3_ are formed rapidly in situ under solidification because V and Cr are strong carbide-forming elements. Nevertheless, Cr atoms could be consumed into the region of VC carbides, and W.T. Wu et al. believed that the Mn atom prefers to occupy the Cr vacancy during solidification [31]. When Mn content increases to above 3.0 wt%, the Cr atom can be replaced by the Mn atom, thus resulting in the reduction or the vanish of Cr_7_C_3_. In addition, compositional undercooling is caused by the Ti elements, where TiC carbide can be produced by the reaction between titanium and carbon due to the lower Gibbs free energy of −183.4 kJ/mol [32]. Subsequently, vanadium and carbon gather gradually to TiC and form VC [33]. As shown in Figure 2 and Figure 10, the corresponding atomic ratio of Ti in the center of the carbides is less than 1 at.%, which indicates that VC can be formed under homogeneous nucleation and the change in free energy can be regarded as the main driving force for in situ carbide particles formation. Surprisingly, the content of Ti elements in the center of the blocky VC particles for the 3.0 Mn and 4.5 Mn samples is greater than 8 at.% based on the intensity of EDS results. The Ti ratio in the center of VC for the 3.0 Mn sample even exceeds 30 at.% when the Ti ratio of other VC particles is still at a low level. Consequently, homogeneous and heterogeneous nucleation can occur simultaneously; however, single heterogeneous nucleation is only present in the 4.5 Mn sample. This indicates that TiC is the heterogeneous nucleation site in the center of the VC particles, and the content of Ti elements within VC increases from the edge to the center [34]. These results reveal that vanadium and carbon elements can gather in the TiC nucleus [35].

Secondly, the effect of Mn content on the shape, size, and distribution of the VC particles is also studied. Figure 2 reveals that the shape of the VC particles changed from the bulky flower-like structure of the 1.5 Mn sample to the tiny blocky structure of the 4.5 Mn sample. This indicates that the Mn element plays an important role in tailoring the shape and size of the VC particles, and the results are in accordance with previous work in reference [36]. Meanwhile, long-distance diffusion of the alloying elements is restrained, resulting in a uniform distribution of alloying elements and carbide; this can be in accordance with the reference [30]. Nevertheless, a weak inhibitory effect exists in the VCFC with low-percentage Mn content and is accompanied by a stronger inhibitory effect in the VCFC with high-percentage Mn content. Thus, the segregation of Cr elements and carbide is only discovered in the 1.5 Mn sample [37], as shown in Figure 2 and Figure 4.

Thirdly, tailoring of the Fe phase in the matrix occurs by turning Mn content. The Mn element plays an important role in the stability of the austenite, which is attributed to Mn partitioning under solidification [38,39,40]. However, the starting temperature of Ms is also reduced by the Mn element; thus, promoting the stability of the austenite [41], as indicated by the XRD patterns (Figure 3) and EBSD (Figure 4) analysis (Figure 8) results. Therefore, a certain amount of austenite is present in the matrix.

### 4.2. Wear Mechanism

It is well known that excellent wear resistance depends on its hardness and friction coefficient [42]. On the one hand, higher hardness can contribute to good wear resistance. Just as in Figure 8, the 3.0 Mn sample has a maximum microhardness; therefore, the corresponding wear rate reaches the minimum value. At the same time, the minimum hardness of the 4.5 Mn sample causes the maximum wear rate. This can be attributed to particulate features and phase constitution. The category, size, shape, and distribution of the carbides can cause a huge difference in hardness and tribological behaviors. A certain amount of homogenous-nucleation VC(HON-VC) and Cr_7_C_3_ carbides exists in the 1.5 Mn sample, just as in Figure 2. However, heterogenous-nucleation VC (HEN-VC) appears in the 3.0 Mn and 4.5Mn samples. Their hardness has a huge difference, as shown in Figure 7. HEN-VC has higher hardness than HON-VC owing to the high-content heterogenous nucleation point of TiC. On the other hand, these carbides are considered as supporting points to prevent serious wear by the counterpart. The carbide with different hardness possesses different carrying capacity. The hardness of Cr_7_C_3_ carbide is less than that of VC carbide; consequently, the average microhardness of the 1.5 Mn sample is lower than that of the 3.0 Mn sample. In particular, phase constitution also transforms from martensite with the BCC structure of the 1.5 Mn sample to austenite with the FCC structure of the 4.5 Mn sample, as shown in Figure 3 and Figure 4. However, the hardness of the matrix for the 3.0 Mn sample is only 7.5 GPa because the C atom is consumed to precipitate VC and TiC carbide. The value is still much greater than free-carbide Fe materials. This manifests that precipitate strengthening and second-phase strengthening can contribute to the hardness of the materials [43,44]. In addition, the size of the carbide is decreased from ~50 μm to ~5 μm, and their distribution in the matrix becomes more and more uniform. During the hardness measurement, the value of the 4.5 Mn sample is relatively low; this can be because the soft phase of the FCC structure exists, and tiny VC carbide collapses under the relatively heavy load.

**Figure 11:**
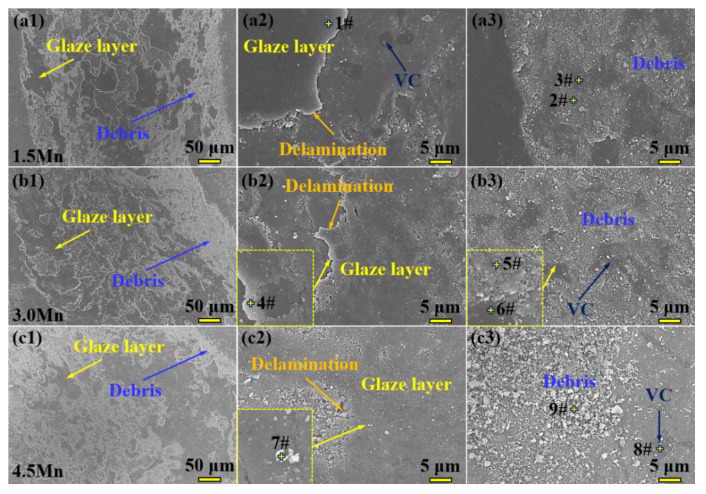
SEM images of wear tracks for three samples. (**a1**–**a3**) 1.5 Mn; (**b1**–**b3**) 3.0 Mn; (**c1**–**c3**) 4.5 Mn.

Just as the aforementioned factors, there is a huge difference in the wear resistance of the three samples. SEM images of the wear tracks for three samples are shown in Figure 11. It is obvious that the area of the glaze layer exhibits increases first and then decreases, and the area of the debris shows a contrary trend. Meanwhile, delamination appears in the wear tracks of all samples. The glaze layer becomes loose, and the size of the debris is bigger and bigger with increasing Mn content. Serious wear occurs in the initial stage because the carbides are unable to retrain the wear of the counterpart, thus resulting in t serious wear of the Fe matrix, and the friction coefficient has a steep climb. At the same time, a large amount of debris is formed and then the carbides are naked to become the main participants. This debris are brought and spread out around the carbides to form a glaze layer; the glaze layer and free debris contain mainly Fe elements with high Si elements. Meanwhile, a higher-content TiC with above 18 at.% exists in the particulate region of the 3.0 Mn sample. A larger area of glaze layer and TiC can contribute to a lower friction coefficient [45]. Afterward, the friction access to the steady stage, wear rate is also decreased, but the counterpart suffers serious wear, as shown in Table 2, the Si content of 5# exceeds 5 at.%. The debris takes part in the process of the friction-wear and forms an intermediate layer, just as this relative friction is stopped between the measured sample and the counterpart. Meanwhile, the wear is also decreased. However, uniformly tiny particles of the Mn sample can be peeled off and attach to the surface of the wear track, as shown in Figure 11(c3); its wear mechanism can be considered typical abrasive wear [46]. On the contrary, bigger particles have a higher supporting capacity, a large proportion of the debris is surrounded around VC particles, and, to form a compact glaze layer, its wear mechanism can be expressed mainly as typical adhesive wear [47].

## 5. Conclusions

In this work, in situ VCp-reinforced, Fe-based composites with different Mn content were prepared by a medium-frequency electromagnetic induction furnace. The influence of Mn elements on the microstructural characteristics of the VC and the texture of the Fe matrix were studied. Furthermore, the influence of microstructure on hardness and tribological behaviors were discussed, some important conclusions were as follows.

1. Mn had a positive effect on adjusting the shape, size, and distribution of VC. The shape of VC was changed from dendrite grains of the 1.5 Mn sample to block grains of the 4.5 Mn sample. In addition, the size of VC particles decreased gradually from ~50 μm to ~5 μm with increasing Mn content.
2. Due to a stronger inhibition effect of Mn on the diffusion rate of other alloying elements and the formation of titanium carbide, their synergistic effect could contribute to nucleation and growth mechanism. With increasing Mn content, the nucleation mechanism was transferred from homogeneous nucleation to heterogeneous nucleation. Furthermore, the addition of Mn also contributed to the reduction of Ms and the stability of retained austenite.
3. With increasing Mn content, hardness could express increase first and then decrease; the maximum value existed in the 3.0 Mn sample, its value is 3.6% and 72.8% higher than that of the 1.5 Mn and 4.5 Mn samples, respectively. Higher microhardness can be attributed mainly to coarse carbides, bcc-structure matrix, and the existence of TiC with higher hardness. However, the FCC-structure matrix and tiny carbide could not withstand a relatively heavy load and thus resulted in the entire collapse and the lower microhardness.
4. Hardness and friction coefficient could contribute to wear resistance. The friction coefficient of the 1.5 Mn, 3.0 Mn, and 4.5 Mn samples were 0.45, 0.38, and 0.48, respectively. Meanwhile, the minimum wear rate of 1.77 × 10^−6^ mm^3^/(N·m) appeared in the 3.0 Mn sample. In addition, the relative wear resistance of the 3.0 Mn sample was 64.4% and 344.1% higher than that of the 1.5 Mn and 4.5 Mn samples. With increasing Mn content, their wear mechanism could be transformed from primary adhesive wear to main abrasive wear.

## Figures and Tables

**Table 1:** Chemical compositions of the VCFCs with different Mn content (wt.%).

Materials	Alloying Elements (wt.%)							
	C	V	Mn	Cr	Si	Mo	Ti	Fe
1.5 Mn	2.8	8.1	1.5	2.5	1.5	1.5	1.5	Bal.
3.0 Mn	2.8	8.1	3.0	2.5	1.5	1.5	1.5	Bal.
4.5 Mn	2.8	8.1	4.5	2.5	1.5	1.5	1.5	Bal.

**Table 2:** The corresponding atomic ratio of 1#~9# in Figure 11.

	Atomic Ratio (at.%)							
	Al	Si	Ti	V	Cr	Mn	Fe	Mo
1#	0.16	1.79	0	4.33	2.66	0.54	89.9	0.59
2#	0.34	3.04	0	2.18	2.48	0.52	90.79	0.62
3#	0	0.97	0.28	71.73	4	0.2	18.7	4.04
4#	0.4	3.26	0	1.39	1.59	1.73	91.27	0.31
5#	0.72	5.22	0.27	2.82	1.02	1.87	87.8	0.27
6#	0	0.1	18.49	76.62	0.5	0.19	2.04	2.07
7#	0	0.88	0	0.73	1.5	0.6	96.11	0.15
8#	0	0	15.38	79	0.57	0.18	2.64	2.09
9#	0.25	2.74	0	0.65	1.6	1.27	93.21	0.23

## Data Availability

The data presented in this study are available on request from the corresponding author.
